# Homochiral Evolution from Self-Assembly of Chiral Block Copolymers

**DOI:** 10.1021/acsmacrolett.6c00449

**Published:** 2026-10-12

**Authors:** Sheng-Wei Shao, Rong-Ming Ho

**Affiliations:** Department of Chemical Engineering, 34881 National Tsing Hua University, No. 101, Section 2, Kuang-Fu Road, Hsinchu 30013, Taiwan, R.O.C.

## Abstract

A block copolymer containing a chiral block is referred to as a chiral block copolymer, which can form a helical phase (H*) in diblock copolymers and an alternating gyroid phase (G^A^) in triblock terpolymers with controlled chirality from self-assembly, as evidenced by electron tomography. Polylactide-containing chiral block copolymers can be used as a representative system for the investigation of homochiral evolution from molecular chirality examined by electronic circular dichroism (ECD) to conformational chirality examined by C=O vibrational circular dichroism (VCD), and finally to phase chirality characterized by electron tomography, with C–O–C VCD providing insight into interchain chiral interactions. The intra- and interchain contributions should be evaluated in the context of the specific molecular structure and sample conditions. By taking advantage of noncovalent chirality induction, it is feasible to introduce a chiral bias by associating a chiral dopant with an achiral block, giving H* with controlled chirality from an achiral block copolymer. This Viewpoint highlights the fundamental principles, providing perspectives on the design of chiral soft materials for device applications.

## Graphical abstract

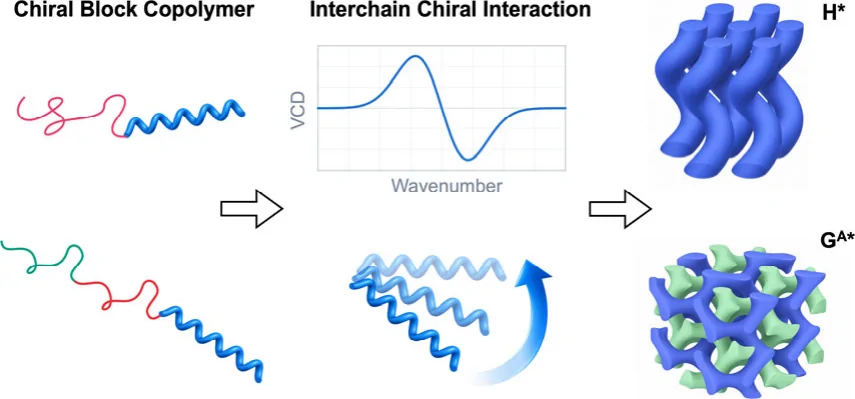

## 1 Chirality Across Multiple Length Scales

Hierarchical chirality can emerge across multiple length scales during self-assembly processes.^1,2^ To understand how chirality propagates and amplifies from the molecular scale to chain conformation, and finally to ordered structures at the mesoscale, we propose a hierarchical framework for chiral self-assembly in polymers, lipids, and surfactants, analogous to the structural hierarchy observed in proteins. In biological systems, protein structures are commonly classified into primary, secondary, tertiary, and quaternary structures, corresponding to amino acid sequence, local conformation, three-dimensional folding, and multichain organization, respectively.^3,4^ By analogy, chirality in polymer systems from self-assembly can also be categorized into several hierarchical levels according to the characteristic length scale and structural organization.^5^ Here, homochiral evolution emphasizes the transfer and amplification of chirality across different structural length scales, rather than the enantiomeric purity of the constituent molecules.

As shown in Figure 1, primary structure, referred to as molecular chirality, originates from the intrinsic stereochemical asymmetry of individual molecules, where a structure is nonsuperimposable on its mirror image, giving rise to enantiomeric forms. Molecular chirality may arise from central chirality, axial chirality and planar chirality.^6^ Secondary structure corresponds to the conformational chirality of a polymer chain, which describes the emergence of a preferred helical conformation or chain twisting at the polymer-chain level. Such chirality is typically associated with the regular arrangement of repeating units along a polymer backbone, leading to a one-handed helical conformation.^7,8^ Tertiary structure represents supramolecular chirality, where chirality emerges from the collective organization of multiple molecules into helical superstructures, including helical fibers, twisted ribbons, and Möbius rings.^9–12^ Quaternary structure corresponds to phase chirality, which describes chirality at the level of a phase possessing long-range periodic order from self-assembly.^5,13,14^ In contrast to supramolecular chirality, phase chirality is defined at the level of periodically ordered chiral structures that can be described by space-group symmetry, such as helical phase (H*) from diblock copolymers and alternating gyroid phase (G^A^) from triblock terpolymers via self-assembly.^5,13–16^ These categories describe different forms of structural organization; note that tertiary supramolecular structures are not necessarily precursors to quaternary phase structures.

**Figure 1:**
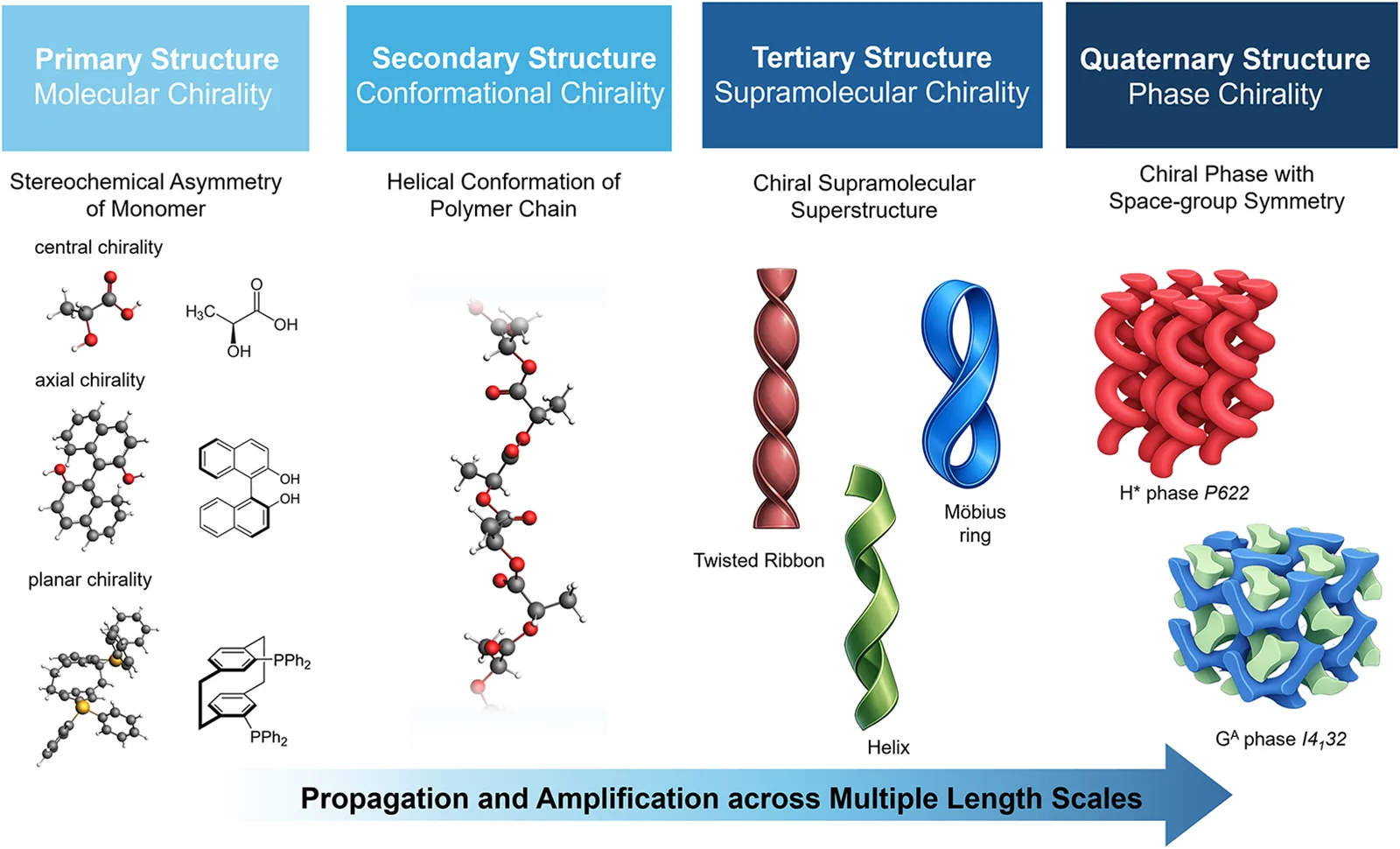
Primary structure originates from the stereochemical asymmetry of the constituent monomers (molecular chirality), including central, axial, and planar chirality. Secondary structure describes the handedness of a polymer chain (conformational chirality), exemplified by a one-handed helical conformation. Tertiary structure represents superstructure chirality generated through the hierarchical assembly of polymer chains into helices, twisted ribbons, and Möbius rings. Quaternary structure corresponds to phase chirality defined by space-group symmetry, as exemplified by hexagonally packed helical phase (H*) with *P*622 symmetry and the alternating gyroid phase (G^A^) with *I*4_1_32 symmetry from block copolymer self-assembly. Together, these structural levels illustrate the propagation and amplification of chirality (homochiral evolution) from molecular building units to macroscopic self-assembled phases. The tertiary structures are not necessarily precursors to the quaternary structures; the latter are distinguished by ordered chiral structures that can be described by space-group symmetry.

Our previous Account and Perspective discussed multiscale chirality transfer, characterization, nanochiral phases, and templated materials.^5,17^ Building on this foundation, the present Viewpoint emphasizes recent developments in gyroid chirality selection, dopant-induced phase chirality, and interfacial chirality transfer, together with unresolved questions about interchain chiral interactions.

## 2 Nanochiral Self-Assembly from Chiral Block Copolymers

Block copolymer self-assembly can generate ordered structures at the nanoscale through microphase separation of chemically distinct polymer blocks. In contrast to polymer blends, where incompatible polymers may undergo macroscopic phase separation, the covalent linkage between different blocks in a block copolymer restricts phase separation to the nanometer scale.^18,19^ As a result, block copolymers with characteristic morphologies such as spheres, cylinders, networks, and lamellae can be self-assembled into well-ordered phases.^20^ The equilibrium phase of conventional block copolymers is generally governed by the segregation strength (χN) and the volume fraction of each block (*f* ^*v*^). The parameter χN describes the degree of thermodynamic incompatibility between blocks, while *f* ^*v*^ determines the preferred interfacial curvature and packing geometry. Therefore, block copolymer self-assembly can be viewed as a balance between minimizing unfavorable interfacial contacts and avoiding excessive chain stretching.

When a block copolymer contains a chiral segment, the block copolymer is referred to as a chiral block copolymer. In such a system, molecular chirality introduces an additional factor beyond the conventional parameters of χN and *f* ^*v*^. When one block possesses stereochemical asymmetry, the chiral block may exhibit a preferred helical chain conformation and chiral interactions between chains during packing. Consequently, chirality can bias the curvature, twist, and handedness of the resulting microdomains, enabling the emergence of chiral phases that are not expected from the phase behaviors of achiral block copolymers.^5,13,14^ Representative examples include H* formed from chiral diblock copolymers (Figures 2a and 2b), and G^A^ formed from chiral triblock terpolymers (Figures 2c and 2d), giving controlled handedness through nanochiral self-assembly.^15,16,21,22^ Here, we use “nanochiral” rather than “mesochiral” to describe chiral self-assembled structures at the nanometer scale, avoiding confusion with achiral meso compounds. In this Viewpoint, we use polylactide-based BCPs as a paradigm to propose a methodology for examining chirality across different structural levels, with particular emphasis on its transfer to phase handedness. Our aim is to provide a framework for investigating these relationships and to encourage further studies in other chiral BCP systems. The broader development of chiral macromolecules and supramolecular assemblies, including their synthesis, characterization, and applications, has been reviewed by Lin and co-workers.^23^ Chiral superstructures formed through solution self-assembly or chiral-additive-induced assembly are also briefly discussed as examples of supramolecular chirality distinct from phase chirality.

**Figure 2:**
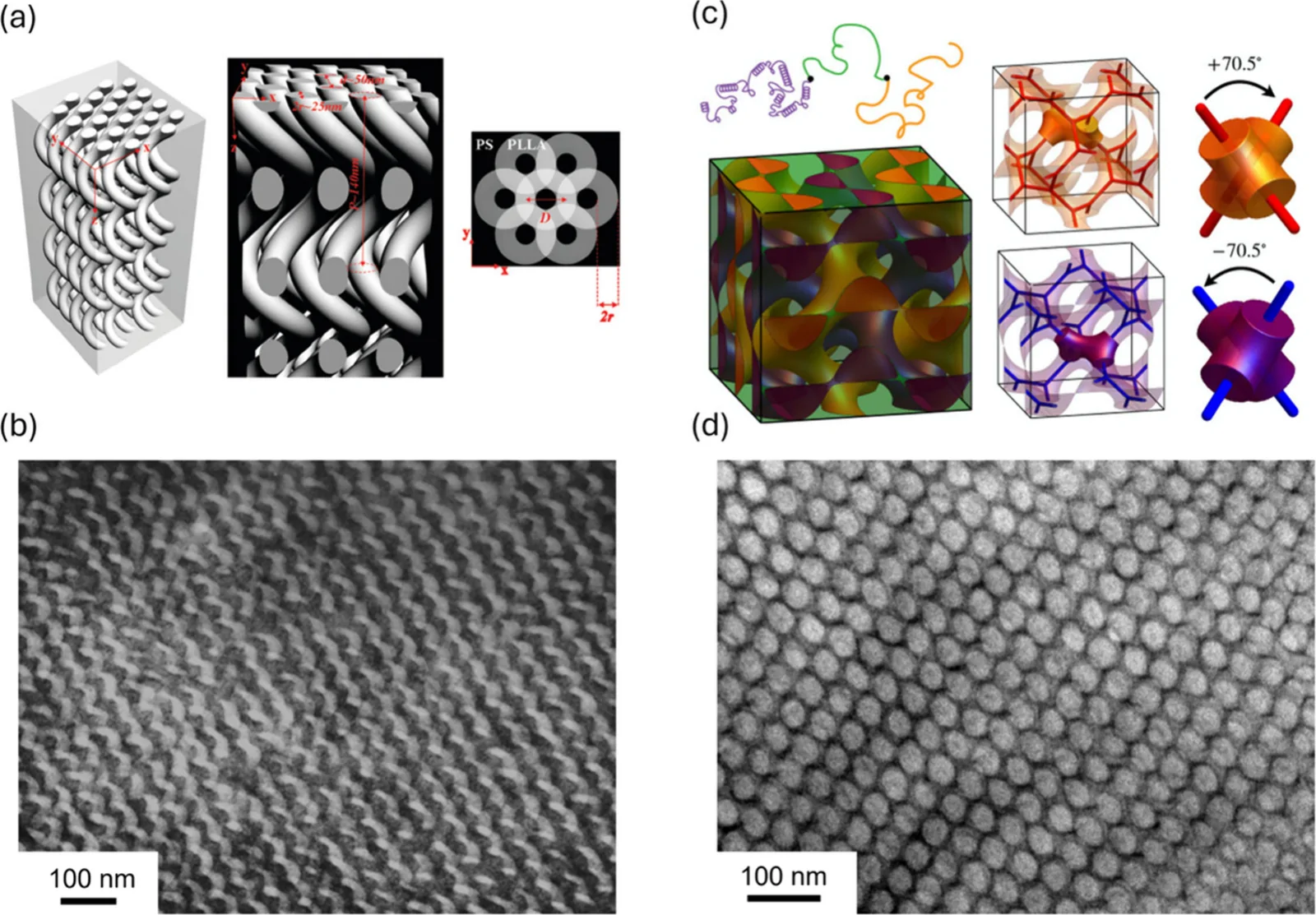
(a) Schematic illustration of a helical phase with a left-handed helix along the helical axis. Reproduced with permission from ref 21. Copyright 2009 American Chemical Society. (b) TEM image of experimentally observed H* from the self-assembly of polystyrene-*block*-poly­(l-lactide) (PS-*b*-PLLA). Reproduced with permission from ref 16. Copyright 2004 American Chemical Society. (c) Schematic illustration of an alternating gyroid network, whose handedness is characterized by the signed dihedral angle between successive network building units. Reprinted with permission from ref 22. (d) TEM image of experimentally observed G^A^ from the self-assembly of polyisoprene-*block*-polystyrene-*block*-poly­(l-lactide) (PI-*b*-PS-*b*-PLLA). Reproduced with permission from ref 15.

## 3 Characterization of Chirality Across Multiple Length Scales

To understand how chirality is transferred and amplified in PS-*b*-PLLA self-assembly, it is necessary to analyze chirality across multiple length scales. Molecular chirality, conformational chirality, supramolecular chirality, and phase chirality represent distinct but interconnected levels of structural asymmetry. In chiral block copolymers, the stereochemical configuration of the chiral block first defines molecular chirality. This local asymmetry may then bias the chain conformation, generate helical conformational preference, influence the packing of polymer chains, and eventually induce symmetry breaking during self-assembly, resulting in the formation of chiral phases. Therefore, the characterization of chiral block copolymers requires a multiscale approach.

### 3.1 Molecular Chirality

At the most fundamental level, molecular chirality refers to the intrinsic asymmetry of individual molecules or repeating units. A molecule is chiral when it is nonsuperimposable on its mirror image, and such chirality may originate from stereogenic centers, axial chirality and planar chirality (Figure 1).^6,24^ In PS-*b*-PLLA, the molecular chirality originates from the stereogenic center in each lactic acid repeating unit (Figure 3a) of the PLLA block. This molecular-level chirality is the primary source of chirality that may subsequently propagate into higher-order structures during block copolymer self-assembly. Molecular chirality is commonly characterized by chiroptical techniques, including electronic circular dichroism (ECD) and vibrational circular dichroism (VCD).^6,25^ ECD probes the differential absorption of left- and right-circularly polarized light associated with electronic transitions and is particularly useful for chiral molecules containing UV–visible chromophores.^6,24^ For example, the molecular chirality of chiral polymers such as PLLA originates from the asymmetric carbon in the lactide repeating unit, and its chiroptical response can be used to distinguish the enantiomeric nature of l-lactic acid and d-lactic acid as shown in Figure 3c.^5,13^ In systems containing stronger chromophores, ECD can further provide structural information through characteristic Cotton effects associated with π-π* or n-π* transitions.^6,24^

**Figure 3:**
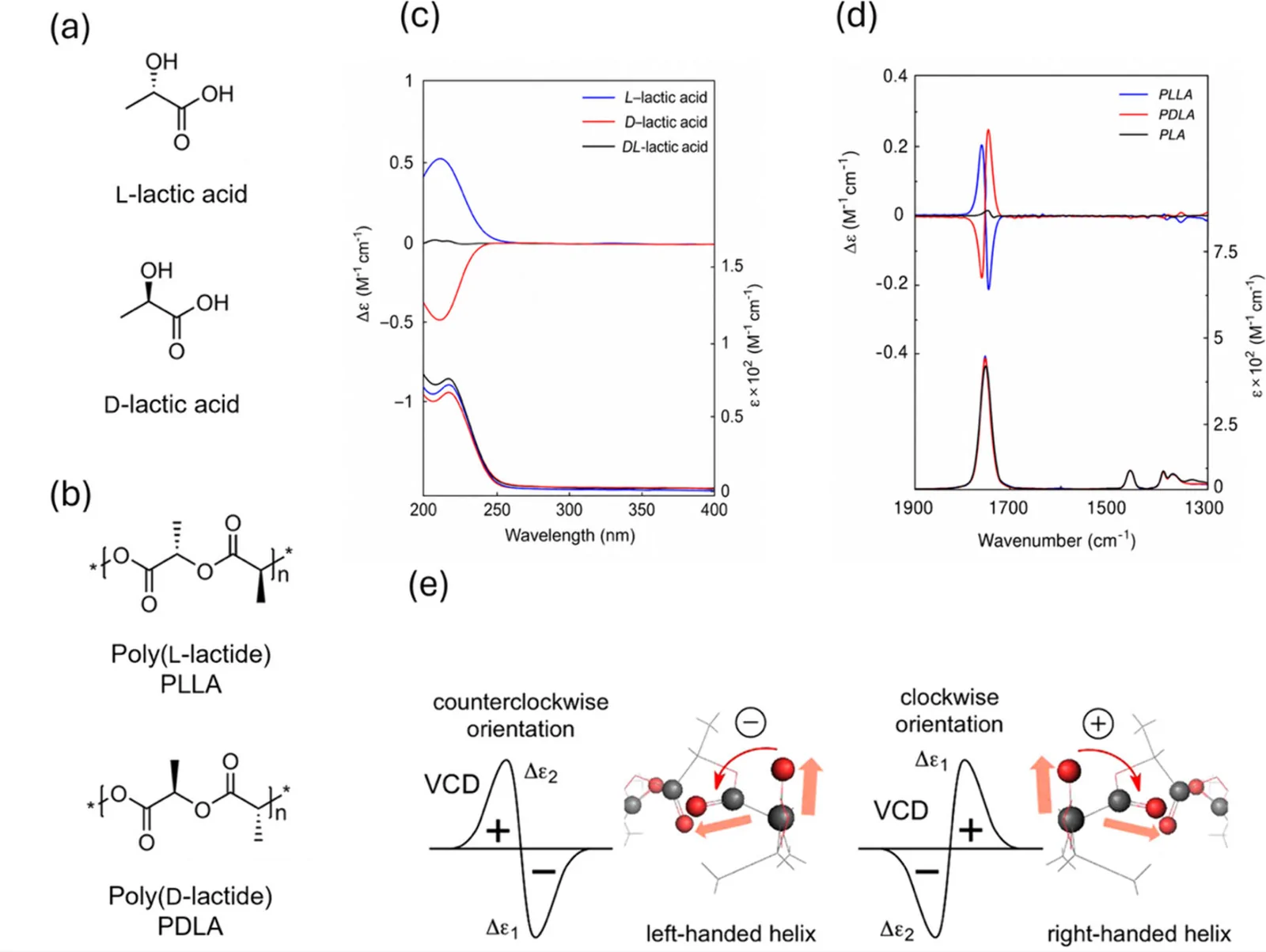
(a) Chemical structures of l-lactic acid and D-lactic acid, illustrating the opposite stereochemical configurations of the chiral monomeric building units. (b) Chemical structures of poly­(l-lactide) (PLLA) and poly­(d-lactide) (PDLA), in which the stereochemical information on the monomers is propagated along the polymer backbone. (c) Electronic circular dichroism (ECD) spectra of (a), showing mirror-image signals associated with the opposite molecular chirality of the two enantiomers. (d) C=O vibrational circular dichroism (VCD) spectra of PLLA and PDLA. Reproduced with permission from ref 13. Copyright 2012 American Chemical Society. (e) Schematic representations of the oppositely handed helical conformations adopted by PLLA and PDLA, exhibiting mirror-image responses arising from the coupling and chiral spatial arrangement of carbonyl-stretching vibrations along the helical polymer chain. Reproduced with permission from ref 31. Copyright 2014 American Chemical Society. Together, ECD and C=O VCD provide evidence of molecular and conformational chirality, respectively, with spectral assignments interpreted in the context of molecular structure and sample conditions.

### 3.2 Conformational Chirality

In contrast to molecular chirality, conformational chirality describes the handedness arising from the three-dimensional arrangement of a polymer chain. In chiral polymers, the regular arrangement of repeating units gives rise to a preferred helical conformation that serves as an intermediate between molecular chirality and higher-order supramolecular chirality or phase chirality. Depending on the polymer structure, the helical sense may be either configurationally stable or dynamically switchable under external stimuli.^7,8,26^ Conformational chirality is commonly characterized by ECD and VCD. While ECD is applicable to polymers containing electronic chromophores, such as conjugated polymers and aromatic helical polymers, VCD is particularly useful for aliphatic polyesters such as PLLA.^7,8,25^ In PLLA (Figure 3b), the C=O stretching vibration is highly sensitive to the relative orientation of neighboring carbonyl groups, making it a useful probe of helical chain conformation when supported by conformational analysis.^13,25–31^ The interpretation of VCD spectra is commonly based on exciton coupling between neighboring transition dipoles. In PLLA, adjacent C=O transition dipoles are oriented approximately perpendicular to the helical axis, giving rise to a characteristic split-type VCD couplet (Figures 3d and 3e).^31^ The opposite helical geometry of PDLA produces a mirror-image spectrum, supporting the conformational interpretation under the conditions examined. However, a bisignate C=O VCD signal alone does not uniquely establish intrachain helicity: lactic acid aggregates also exhibit such responses influenced by hydrogen bonding and molecular association.^32^ Unlike lactic acid, PLLA repeat units contain ester linkages rather than free hydroxyl and carboxylic acid groups. The relative contributions of intra- and intermolecular interactions should therefore be evaluated for the specific molecular structure and sample conditions, alongside structural characterization and appropriate calculations. Both ECD and VCD measurements of solid samples require particular care, because linear dichroism and linear birefringence can introduce artifacts into the measured spectra. Sample rotation and front-back measurements, together with assessment of linear anisotropy, help evaluate these contributions.^33^

### 3.3 Supramolecular Chirality

In contrast to molecular and conformational chirality, supramolecular chirality emerges from the collective organization of polymer chains into chiral nanostructures. At this hierarchical level, handedness is determined by the spatial arrangement of multiple molecular building blocks rather than by the stereochemistry of individual repeating units or the conformation of a single polymer chain. Representative supramolecular morphologies include twisted ribbons, helical fibers and Möbius strips.^7–9^ A representative example is provided by polylactide-containing block copolymers, in which chiral PS-*b*-PLLA or PS-*b*-PDLA can form Möbius-strip-like rings through self-assembly in solution.^11,34^ The formation of these topologically chiral objects demonstrates that molecular and conformational chirality can be further amplified into discrete supramolecular structures without requiring these structures to precede long-range phase chirality. Supramolecular chirality is primarily characterized by real-space imaging techniques, including atomic force microscopy (AFM), scanning electron microscopy (SEM), and transmission electron microscopy (TEM), which directly resolve the handedness, twist, curvature, and topology of these self-assembled nanostructures. For example, AFM was used to characterize the morphology and helical sense of azobenzene-containing BCP nanowires, including helicity inversion upon varying the degree of polymerization.^35^ Complementary spectroscopic analysis is illustrated by supramolecular assemblies of N-centered triarylamine trisamides (TATA): combined ECD, VCD, and calculations distinguished changes in individual fibril helicity from the handedness of the overall fibril assembly.^36^

### 3.4 Phase Chirality

Phase chirality refers to chirality expressed in long-range ordered phases with defined symmetry and three-dimensional spatial arrangement. In chiral block copolymer self-assembly, representative examples include the helical phase (H*) in chiral diblock copolymers and the alternating gyroid phase (G^A^) in chiral triblock terpolymers.^5,13–15,37^ The structural origin and self-assembly mechanism of these chiral phases will be discussed in later sections; here, we focus mainly on how phase chirality can be characterized. For the H* phase, two-dimensional TEM provides important real-space information on the projected morphology through its characteristic crescent-like projection images. However, because a TEM image represents a two-dimensional projection of a three-dimensional structure, it is insufficient to determine the handedness unambiguously. As illustrated in Figure 4a, the projected images of left- and right-handed H* structures are essentially indistinguishable because the out-of-plane helical trajectories are collapsed onto the same projection plane.^5^ This limitation becomes even more pronounced for structurally complex chiral morphologies such as gyroid networks, in which handedness is encoded in the three-dimensional connectivity of the network rather than in any single projected image.^13,15^ Electron tomography is therefore required to reconstruct the three-dimensional morphology of the H*, allowing the helical diameter, pitch, and handedness to be directly visualized. As shown in Figure 4b, the reconstructed volumes reveal continuous helical trajectories and distinguish the handedness of H* through their senses of rotation.^13^ In addition to structural imaging, VCD spectroscopy associated with C–O–C vibrations provides complementary information on interchain chiral packing. The transition dipole moment of the C–O–C vibration can be approximately parallel to the polymer chain axis (Figure 4c). Consequently, its VCD response (Figure 4d) is highly sensitive to the relative packing of neighboring polymer chains, and thus provides complementary evidence of intermolecular chiral organization within the H*, with contributions evaluated alongside conformation, crystallinity, and packing. For the G^A^ phase, phase chirality is associated with the handed three-dimensional connectivity of the gyroid network. Since opposite-handed gyroid networks can appear similar in two-dimensional projections, three-dimensional reconstruction is essential for determining the actual handedness.^15^

**Figure 4:**
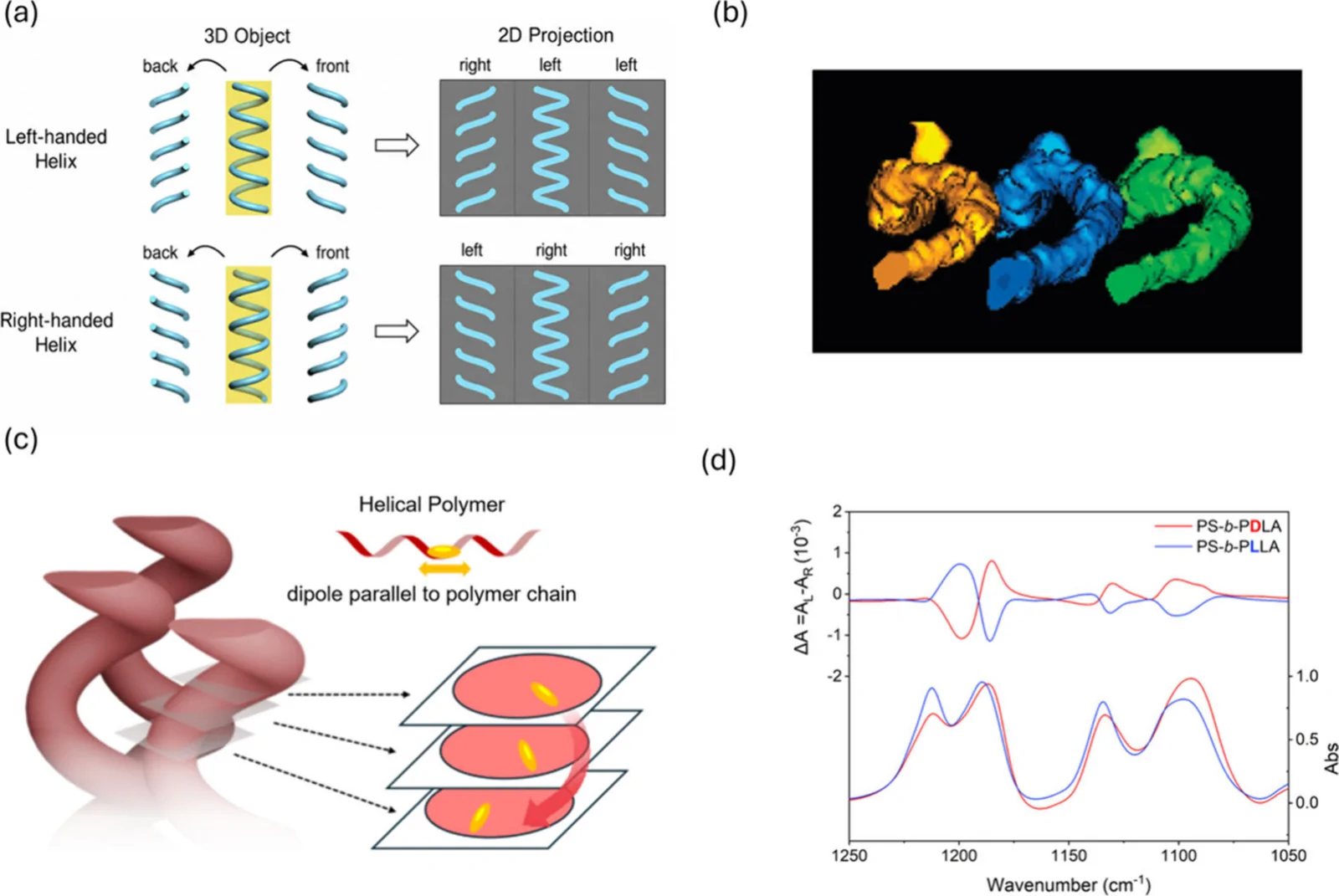
(a) Schematic illustration comparing three-dimensional left- and right-handed helical structures with their corresponding two-dimensional projection images, demonstrating that opposite-handed helices can produce indistinguishable TEM projections depending on the viewing direction. Reproduced with permission from ref 5. Copyright 2017 American Chemical Society. (b) Three-dimensional reconstruction obtained by electron tomography, revealing the helical morphology of the microdomains. Reproduced with permission from ref 21. Copyright 2009 American Chemical Society. (c) Schematic representation of the C–O–C transition dipole moments, showing their predominant orientation parallel to the polymer-chain axis and their sensitivity to interchain chiral packing. (d) VCD and corresponding IR spectra in the C–O–C vibration region of self-assembled H* from PS-*b*-PLLA and PS-*b*-PDLA, exhibiting mirror-image VCD responses associated with opposite phase chirality.

## 4 Homochiral Evolution in Chiral Block Copolymer Self-Assembly

The hierarchical characterization discussed in the previous section establishes the structural framework for understanding chirality across multiple length scales. A central question, however, is how molecular chirality propagates through conformational organization and intermolecular interaction to ultimately generate long-range phase chirality during block copolymer self-assembly. This transfer and amplification of chiral information, referred to here as homochiral evolution, underlies the emergence of phase chirality that cannot be explained by conventional block copolymer thermodynamics alone. In this section, we discuss the theoretical frameworks, experimental evidence, and basic principles governing homochiral evolution in chiral block copolymers, highlighting the formation of H* and G^A^ with controlled chirality.

### 4.1 Theoretical Prediction for H* from Chiral Block Copolymers*

The discovery of the H* in chiral block copolymers provided the first experimental evidence that chirality could be amplified into a periodically ordered chiral phase through block copolymer self-assembly. Early studies of PS-*b*-PLLA demonstrated the formation of H*. However, subsequent studies showed that the H* gradually transformed into a conventional double-gyroid phase upon prolonged thermal annealing, suggesting that the experimentally observed H* from the PS-*b*-PLLA is not always the thermodynamic equilibrium state but could instead represent a metastable intermediate.^38^ This raised an important question: can chirality alone stabilize a chiral phase at thermodynamic equilibrium, or is the H* merely a kinetically trapped metastable phase? Conventional self-consistent field theory (SCFT) successfully predicts equilibrium phases from block copolymer self-assembly by balancing interfacial energy and chain entropy as functions of the segregation strength (χN) and block composition (*f* ^*v*^).^39,40^ However, conventional SCFT cannot capture the orientational preference of chiral polymers and therefore fails to predict intrinsically chiral phases since polymer segments are treated as isotropic Gaussian chains. To address this limitation, Grason and co-workers developed an orientational self-consistent field (oSCF) theory that incorporates a vector order parameter describing local chain orientation together with a Frank-type chiral interaction favoring cholesteric twist (Figure 5a).^41,42^ Consequently, the phase diagram is governed not only by χN and *f* ^*v*^ but also by the strength of chiral interactions. Composition controls microdomain proportions and interfacial curvature, whereas χN controls segregation; molecular weight also affects domain size and relaxation, and polymer architecture constrains network connectivity. Together with chiral interaction strength, these factors determine morphology selection. The H*-to-double-gyroid transition in PS-*b*-PLLA also illustrates how solvent-removal conditions and thermal annealing affect metastability and the observed morphologies.^38^

**Figure 5:**
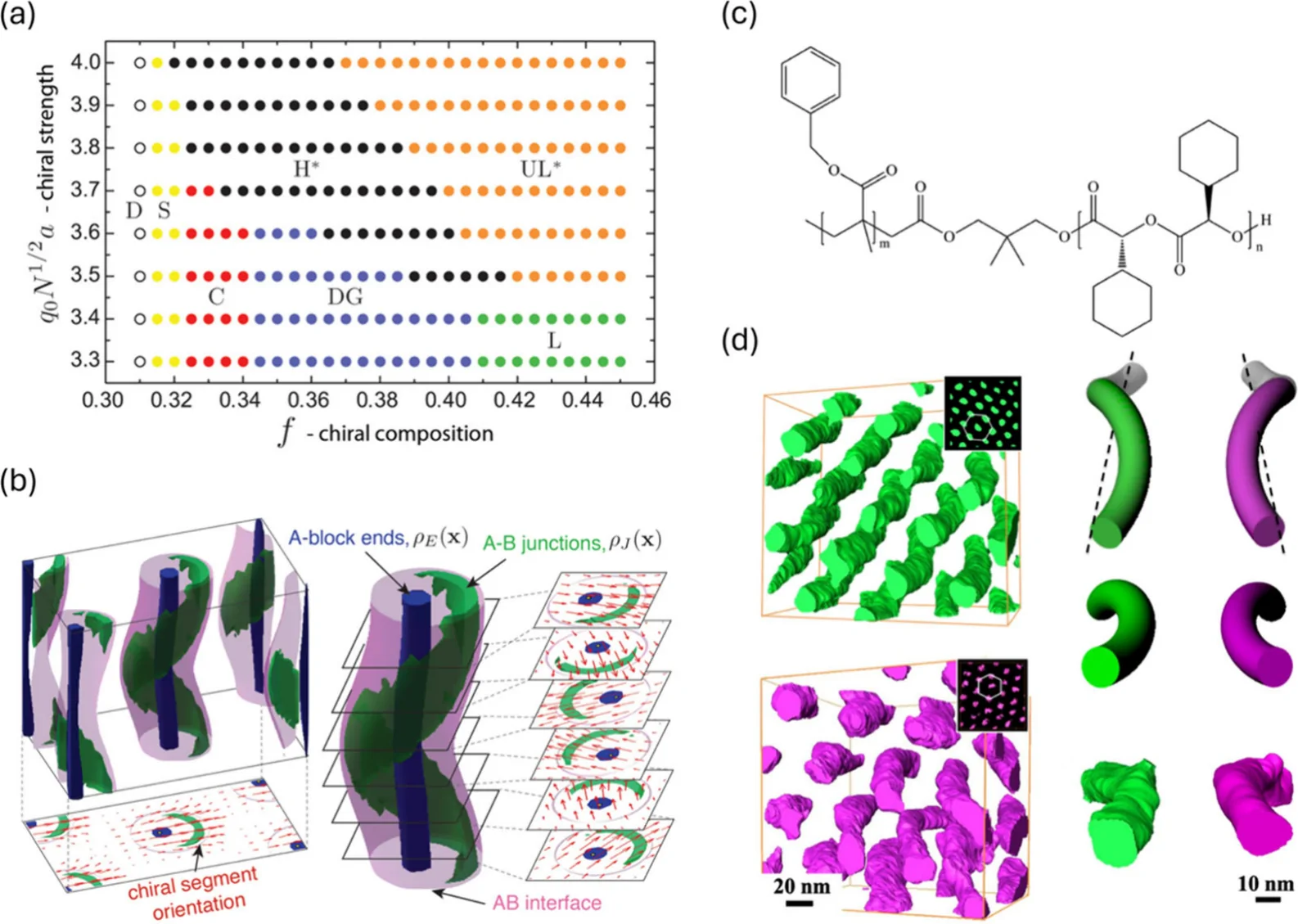
(a) Orientational self-consistent field (oSCF) theory phase diagram for chiral block copolymers at fixed segregation strength (χ*N* = 14), showing that increasing chiral interaction stabilizes two chiral phases: helical phase (H*) and undulated lamellar phase (UL*), in addition to conventional lamellar (L), cylindrical (C), spherical (S), and double gyroid (DG) phases. Reprinted with permission from ref 41. Copyright 2013 by the American Physical Society. (b) Prediction of the equilibrium H* from the oSCF theory, illustrating the helical rotation of cylindrical microdomains accompanied by a cholesteric twist of chiral segment orientation along the microdomain axis. Reproduced with permission from ref 14. Copyright 2015 American Chemical Society. (c) Chemical structure of poly­(benzyl methacrylate)-*b*-poly­(*D*-cyclohexylglycolide) (PBnMA-*b*-PDCG), designed to enhance chiral interactions through bulky chiral side groups while maintaining a segregation strength comparable to that of PS-*b*-PLLA. (d) Three-dimensional electron tomography of left- and right-handed H* phases formed by PBnMA-*b*-PLCG (top) and PBnMA-*b*-PDCG (bottom), respectively. Insets show the hexagonal packing of helical cylinders viewed along the helical axis, confirming the experimentally realized phase chirality. Reproduced from ref 43.

The oSCF calculations predict two equilibrium chiral phases that are absent from the phase diagram of conventional block copolymers: the helical phase (H*) and the undulated lamellar phase (UL*) (Figure 5a).^41^ Among these, the H* phase is particularly significant because it represents the first equilibrium morphology in which cylindrical microdomains collectively adopt one-handed helices while preserving long-range hexagonal order. This behavior originates from the competition between chain stretching and chiral orientational interactions. Although straight cylindrical microdomains minimize chain stretching, they cannot accommodate the preferred cholesteric twist of neighboring chiral segments. Beyond a critical chiral interaction strength, the cylindrical microdomains spontaneously deform into helices, partially relieving orientational frustration while maintaining the microphase-separated interface (Figure 5b). More importantly, oSCF theory establishes the first thermodynamic framework linking molecular chirality to phase chirality, demonstrating that molecular chirality can reshape the equilibrium free-energy landscape rather than merely perturb conventional block copolymer self-assembly. This theoretical framework laid the foundation for understanding homochiral evolution and motivates the subsequent experimental realization of thermodynamically stable H*.^43^

### 4.2 Stable H* Phase and Generality in Chiral Block Copolymers

Although oSCF theory predicted that the H* could exist as a thermodynamically stable equilibrium morphology, experimental verification remains challenging because the H* initially observed in PS-*b*-PLLA was identified as a metastable phase, suggesting that the chiral interactions are insufficient to stabilize the equilibrium H*. To enhance the strength of chiral interactions while maintaining comparable microphase-separation behavior, Ho and co-workers designed methacrylate-based block copolymers containing poly­(D-cyclohexylglycolide) (PDCG) as the chiral block (Figure 5c). Under appropriate annealing conditions, PBnMA-*b*-PDCG reproducibly forms H* as its equilibrium morphology even after one month of thermal annealing. Three-dimensional electron tomography revealed continuous one-handed helical cylinders arranged on a long-range hexagonal lattice (Figure 5d), in excellent agreement with the structures predicted by oSCF theory. These results establish the H* as a genuine thermodynamic equilibrium phase and demonstrate that sufficiently strong chiral interactions can stabilize the resulting chiral phase.^43^ Subsequent studies further revealed that this behavior is not unique to a specific polymer chemistry. In addition to PS-*b*-PLLA and methacrylate-based block copolymers containing PDCG, similar helical morphologies have also been reported in poly­(4-vinylpyridine)-*block*-poly­(l-lactide) (P4VP-*b*-PLLA).^44^ Despite their distinct molecular structures, these systems suggest a common principle: molecular chirality first induces a preferred local chain orientation, which subsequently biases intermolecular packing during self-assembly. Collectively, these studies demonstrate that the emergence of phase chirality is consistent with a common thermodynamic picture whose quantitative balance depends on polymer chemistry. Whether chirality originates from chiral polymer backbones, bulky chiral side groups, or supramolecular interactions, chiral interactions can modify the equilibrium free-energy landscape and favor chiral phases under suitable conditions. This principle suggests a design strategy for engineering morphologies through the self-assembly of chiral block copolymers and provides the experimental foundation for extending homochiral evolution from one-dimensional helical phases to more complex three-dimensional bicontinuous networks.

### 4.3 Gyroid with Controlled Chirality in Chiral Triblock Terpolymers

The formation of nanochiral phases suggests that phase chirality should also extend to more complex three-dimensional network morphologies. Among these network morphologies, the alternating gyroid (G^A^) illustrates how phase chirality arises from the three-dimensional arrangement and symmetry of chemically distinct networks. An ideal conventional double gyroid consists of two interpenetrating, chemically equivalent networks of opposite handedness and is therefore morphologically achiral. In an alternating gyroid, the two opposite-handed networks are occupied by different blocks, removing the symmetry that exchanges them and giving rise to a chiral morphology.^15^ Selective removal of one network-forming block, followed by templated synthesis within the resulting nanochannels, enables the fabrication of a single-gyroid replica.^45,46^ However, a chiral structure within an individual grain does not necessarily imply a common handedness throughout the sample, because grains of opposite handedness may coexist. Consequently, the G^A^ phase provides an ideal platform for investigating whether molecular chirality can be transferred into higher-dimensional periodic structures.^15^ Using chiral triblock terpolymers, Ho and co-workers demonstrated that the handedness of the gyroid network can be precisely controlled by the molecular chirality of the polylactide block.^15^ PI-*b*-PS-*b*-PLLA and PI-*b*-PS-*b*-PDLA form mirror-image alternating gyroid networks, whereas the corresponding achiral PI-*b*-PS-*b*-PLA self-assembles into an achiral alternating diamond network (Figures 6a-f). Three-dimensional electron tomography directly reconstructed the interconnected PI networks and confirmed the opposite handedness of the two enantiomorphic gyroid structures. To further validate the reconstructed morphologies, the polylactide domains were selectively replicated with nickel to produce freestanding metallic networks that preserved the original topology (Figures 6g-i). FESEM observations along the [111] direction reveal left- and right-handed helical features in the Ni networks replicated from the PLLA and PDLA domains, respectively (Figures 6g and h). These Ni networks exhibit handedness opposite to that of the corresponding PI networks reconstructed along [110], consistent with the alternating-gyroid morphology. Unlike the H*, where handedness can be directly identified from the helical trajectory of cylindrical microdomains, the chirality of a gyroid network is embedded in the three-dimensional connectivity of the network. Consequently, quantitative geometric descriptors are required to distinguish opposite-handed structures. A particularly effective approach is the signed dihedral-angle analysis proposed by Grason and co-workers (Figure 6j).^47^ By defining the dihedral angle between consecutive trigonal normals along the gyroid network, the handedness of the network can be quantified by the sign of the dihedral angle. Statistical analysis of the dihedral-angle distributions revealed mirror-image polar distributions for PI-*b*-PS-*b*-PLLA and PI-*b*-PS-*b*-PDLA (Figures 6k and 6l), providing a robust and quantitative descriptor to distinguish left- and right-handed gyroid networks.^15^ Grason and co-workers related chirality selection in alternating gyroid networks to biaxial segmental order and twist.^22^ This analysis concerns handedness selection within a network morphology, complementing the effects of composition, segregation strength, architecture, and chiral interaction strength on morphology selection. The connection between interchain organization and network chirality is further illustrated by studies of double-gyroid symmetry breaking and network transitions.

**Figure 6:**
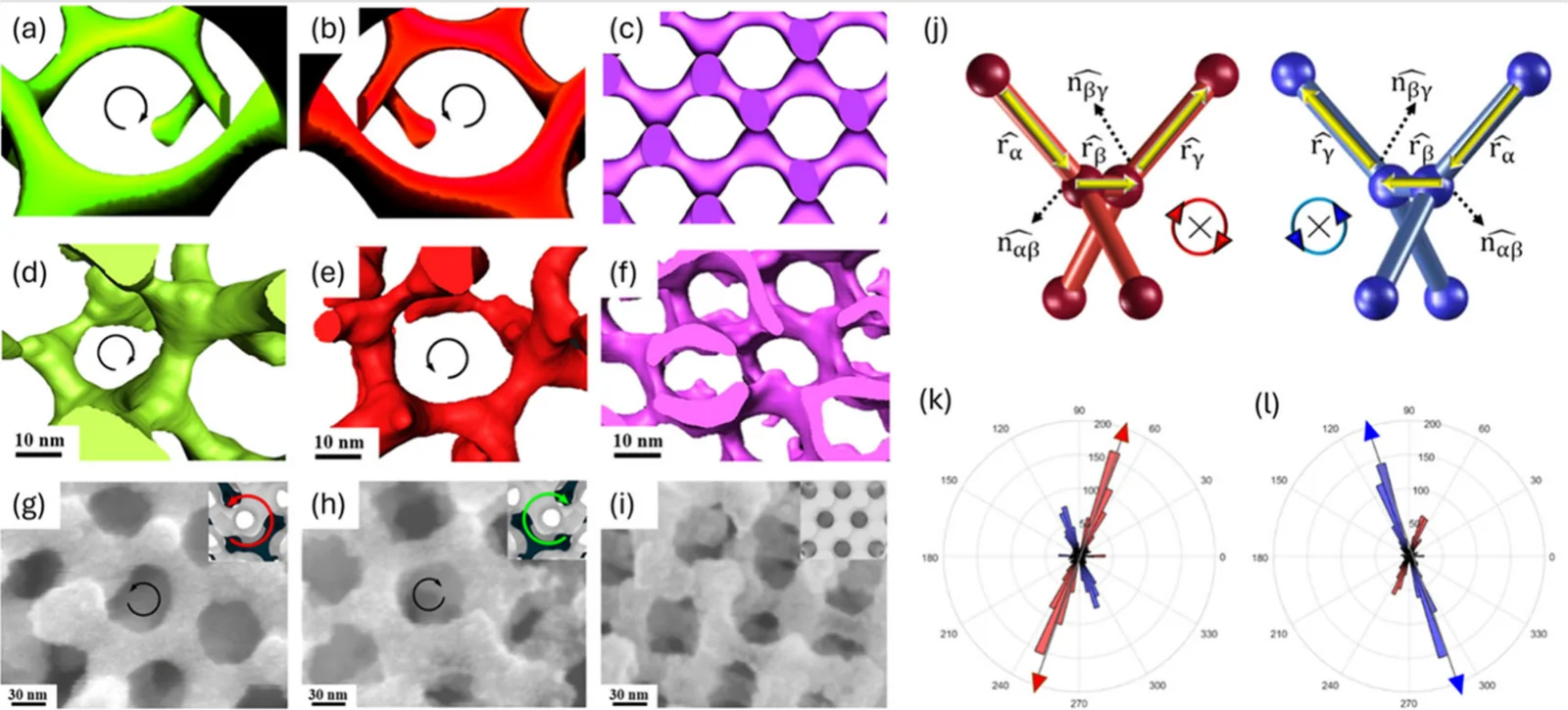
(a-c) Simulated [110] projections of the PI network for PI-*b*-PS-*b*-PLLA, PI-*b*-PS-*b*-PDLA, and PI-*b*-PS-*b*-PLA, respectively. (d-f) Three-dimensional electron tomography reconstructions of the corresponding networks, revealing right-handed and left-handed gyroid networks for the PLLA- and PDLA-containing triblock copolymers, respectively, whereas the achiral system forms an achiral diamond network. (g-i) FESEM images of the replicated Ni networks after selective removal of the polylactide microdomains. The handedness observed along the [111] direction agrees with the simulated structures shown in the insets. (j) Definition of the signed dihedral angle for the handedness of the gyroid network based on the rotational angle between two consecutive trigonal normals. (k,l) Polar distributions of the dihedral angles for PI-*b*-PS-*b*-PLLA and PI-*b*-PS-*b*-PDLA, respectively, demonstrating opposite distributions that quantitatively distinguish the two enantiomorphic gyroid networks. Reproduced with permission from ref 15.

Our previous studies further illustrate the relationship between interchain organization and network chirality. We reported residual VCD enhancement in a double-gyroid phase, supported by oSCF calculations predicting a weak volume asymmetry between opposite-handed networks above a critical chiral strength, consistent with a chiral double gyroid (DG*).^48^ We subsequently examined composition-dependent transitions between alternating gyroid and core–shell double-gyroid phases in chiral triblock terpolymers. Differences in the C–O–C VCD response were interpreted in terms of interchain chiral interactions and the packing of helical chains within these networks.^49^ As a result, these studies highlight the importance of relating interchain organization to network symmetry and the resulting chiroptical response. These results demonstrate that homochiral evolution is not restricted to one-dimensional helical phases but can also generate well-defined phase chirality in three-dimensional bicontinuous networks while preserving long-range crystallographic order. More importantly, the combination of three-dimensional electron tomography and quantitative geometric analysis establishes a general framework to identify phase chirality in complex self-assembled nanostructures.

## 5 Induced Chirality in Achiral Block Copolymers

Unlike chiral block copolymers, where chirality is encoded intrinsically within the polymer backbone, achiral block copolymers provide an alternative strategy to generate chiral nanostructures through external chiral induction. In these systems, molecular chirality is introduced by chiral dopants or supramolecular interactions rather than covalent incorporation into the polymer chain. Despite the absence of inherent stereogenic centers in the block copolymer itself, the transferred chiral information can still propagate across multiple hierarchical length scales, ultimately giving rise to a chiral phase with preferred chirality. This phenomenon demonstrates that phase chirality is governed not only by the chemical structure of the polymer but also by the ability of molecular chiral information to couple with block copolymer self-assembly.^7,8,26,50–54^ In contrast to chiral block copolymers, induced chirality provides considerably greater flexibility because the handedness can be controlled simply by selecting the appropriate chiral inducer without requiring new polymer synthesis. Consequently, induced chirality has emerged as a versatile strategy for manipulating block copolymer self-assembly and further demonstrates that chirality amplification is a general physical phenomenon rather than an intrinsic property of specific polymers.

### 5.1 Chirality Transfer from Small Molecules to Helical Polymer Chains

Is there a simple approach to creating helical polymer chains with preferred handedness? The concept of inducing chirality in achiral polymers through external chiral molecules has been extensively developed by Yashima and co-workers,^7,8,26,54^ who established a general strategy for chirality transfer and amplification in dynamic helical polymers. Rather than relying on covalently incorporated stereogenic centers, these systems utilize noncovalent interactions between achiral polymers and enantiomerically pure small molecules to generate a preference for one helical handedness.^8^ Because the polymer backbone remains conformationally dynamic, a small chiral bias introduced by guest molecules can be cooperatively amplified along the entire polymer chain, leading to a pronounced macromolecular helicity and strong chiroptical responses. Upon association with chiral guests through hydrogen bonding, ionic interactions, or other noncovalent forces, neighboring repeating units collectively adopt the same helical sense, producing a large induced circular dichroism (ICD) signal even when the amount of chiral inducer is extremely small. This cooperative mechanism enables dynamic helical polymers to function as highly sensitive chirality sensors capable of detecting minute enantiomeric imbalances in solution.^26^ More importantly, these studies established the fundamental physical principle that molecular chirality introduced through supramolecular interactions can be cooperatively amplified into macromolecular chirality without covalent modification of the polymer backbone.

### 5.2 Induced H* Phase in Achiral Diblock Copolymers

Building on Yashima and co-workers’ concept of chirality induction in dynamic helical polymers,^7,8,26^ achiral block copolymers can be induced to exhibit phase behavior similar to that of chiral block copolymers, enabling the formation of chiral phases through microphase separation. As shown in Figure 7, the formation of H* can be achieved by using cylinder-forming polystyrene-*block*-poly­(ethylene oxide) (PS-*b*-PEO) for association of chiral dopant BINOL with achiral PEO through hydrogen bonding; the interchain chiral interaction from the induced chirality of polymer chains drives a phase transition from the conventional HC to the H*.^53^ The emergence of the H* dramatically amplifies the chiroptical responses of both the polymer host and the molecular guest. The ordered helical packing strengthens interchain chiral interaction, giving rise to pronounced ICD of the PEO block and enhanced circularly polarized luminescence (CPL) from the associated BINOL molecules.^53^ More importantly, this study suggests a conceptual strategy that combines chirality induction in dynamic helical polymers with the microphase separation of block copolymers. Instead of requiring the dedicated synthesis of chiral block copolymers, molecular chirality transferred from chiral dopants is cooperatively amplified through microphase separation, resulting in the formation of a chiral phase. A related example is the coassembly of achiral star-shaped PAA-*b*-PS unimolecular micelles with R- or S-mandelic acid reported by Lin and co-workers. Hydrogen bonding mediates chirality transfer to hierarchical fibrillar assemblies, which exhibit strong, invertible chiroptical responses and enable circularly polarized luminescence from incorporated achiral emitters.^55^ Within the framework used here, this example illustrates induced supramolecular chirality; by contrast, establishing phase chirality additionally requires characterization of periodic organization and its symmetry.

**Figure 7:**
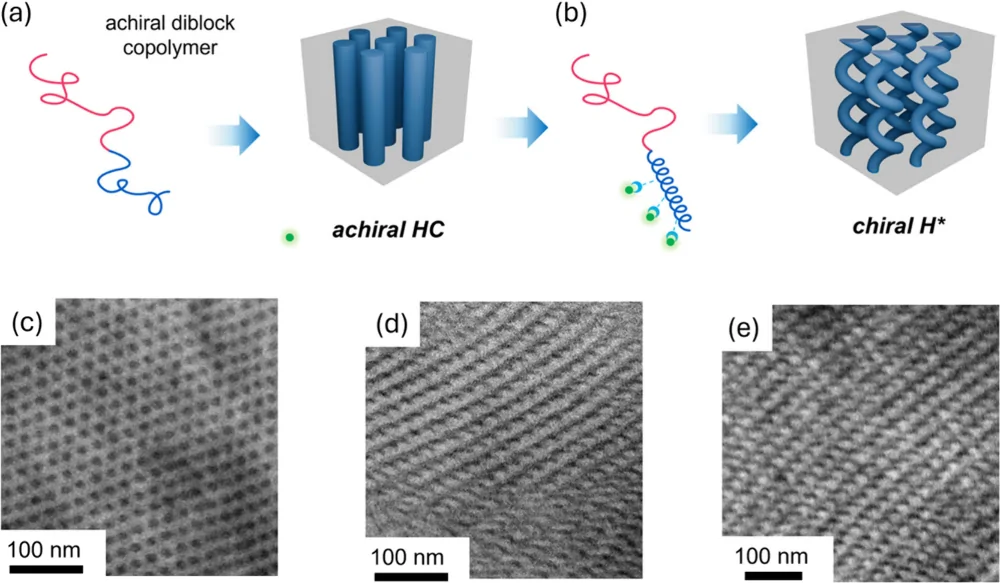
(a) Schematic illustration of an achiral diblock copolymer self-assembling into an achiral hexagonally packed cylinder (HC) phase. (b) Upon association with a chiral dopant through noncovalent interaction, the HC phase transforms into a helical phase (H*). (c-e) TEM micrographs of neat PS-*b*-PEO and PS-*b*-PEO associated with (*R*)-BINOL and (*S*)-BINOL, respectively. Reproduced from ref 53 under a CC BY 4.0 license.

The successful induction of H* in achiral diblock copolymers naturally raises the question of whether externally supplied molecular chirality can also be transferred into more complex three-dimensional network morphologies. As discussed in the previous section, intrinsically chiral triblock terpolymers can self-assemble into G^A^ phases with controlled chirality through homochiral evolution.^15^ Whether dopant-induced conformational chirality can similarly select the handedness of an alternating gyroid in an achiral triblock terpolymer remains a question for future investigation.

## 6 Chiral Metamaterials

Chiral metamaterials are artificial structures with engineered geometries that interact differently with left- and right-circularly polarized light, enabling optical properties rarely found in naturally occurring materials. Their unique chiroptical responses have attracted considerable interest for applications in circular polarization control, chiral sensing, optical communication, negative refraction, and photonic devices.^56,57^ Conventionally, many optical chiral metamaterials, particularly three-dimensional plasmonic metamaterials, have been fabricated using top-down nanofabrication methods such as electron-beam lithography, focused-ion-beam processing, and direct laser writing.^56,58–60^ Although these approaches provide excellent structural precision, their fabrication cost, throughput, and accessible area depend on the method and structural complexity, particularly for complex three-dimensional architectures with nanoscale feature sizes.^46,61^ In contrast, block copolymer self-assembly offers a versatile bottom-up strategy for constructing periodic structures as solid phases; most interestingly, by taking advantage of templated synthesis,^61^ it is possible to create a variety of chiral inorganic network materials at the nanoscale. Selective degradation of one constituent block in a self-assembled chiral phase produces a nanoporous polymer with continuous nanochannels. This porous structure can then serve as a template for replicating the original morphology in organic or inorganic materials. With the use of chiral templates possessing characteristic dimensions of tens of nanometers, it is possible to create three-dimensional chiral metamaterials with chiroptical functionalities through preservation of the chiral geometry. BCP self-assembly offers a potentially scalable fabrication route; however, large-area structural uniformity and replication fidelity during metal deposition remain important challenges.

### 6.1 Chiral Plasmonic Metamaterials

Plasmonic nanostructures are particularly attractive because their localized and coupled plasmon modes can produce pronounced differential interactions with left- and right-circularly polarized light in the visible and near-infrared regions.^56^ A representative example is the fabrication of chiral plasmonic films using the self-assembled H* followed by degradation of one constituent block as a template. As shown in Figure 8a, self-assembled PS-*b*-PLLA and PS-*b*-PDLA films containing one-handed helical cylinders are first converted into nanoporous PS through selective hydrolysis of the polylactide microdomains. Subsequently, templated electroless plating can be carried out to produce Au nanohelices with controlled handedness embedded within the PS matrix (Figures 8b and 8c). The handedness of the H* template is faithfully inherited by the deposition of Au, giving rise to experimentally observed mirror-image circular dichroism spectra that can be reproduced by finite-difference time-domain simulations (Figures 8d and 8e). In this case, the optical activity originates from the individual metallic helices with controlled handedness, giving the associated plasmonic eigenmodes that correspond to the direct transfer of phase chirality from the polymer template to discrete chiral plasmonic nanoobjects.^62^

**Figure 8:**
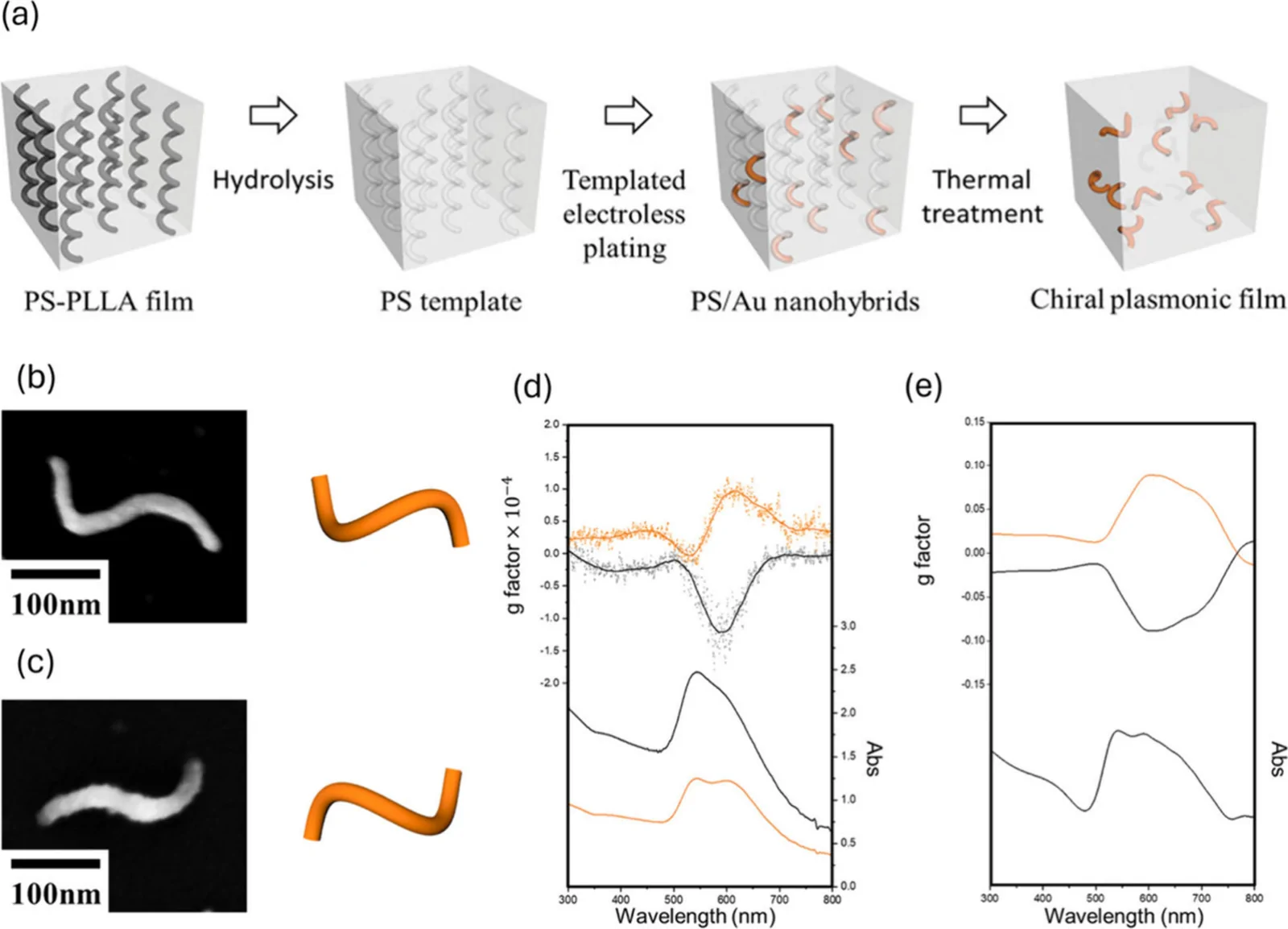
(a) Schematic illustration of the fabrication of chiral plasmonic PS/Au nanohybrid films containing gold nanohelices by templated electroless plating. (b,c) TEM micrographs and corresponding idealized models of left-handed and right-handed Au nanohelices fabricated from PS-*b*-PLLA and PS-*b*-PDLA templates, respectively. (d,e) Experimental (d) and FDTD-simulated (e) g-factor spectra (upper panels) and corresponding absorption spectra (lower panels) of the Au nanohelices. Reproduced with permission from ref 62. Copyright 2020 Wiley-VCH GmbH.

The demonstrated chiroptical performance remains limited, with experimental g-factors of approximately 10^–4^ for these Au nanohelix films. Comparisons with other chiral plasmonic structures should account for material, geometry, orientation, and the measured optical quantity.^63^ Note that Kotov and co-workers reported chiral Au nanoparticles synthesized under circularly polarized illumination with g-factors of up to 0.4.^64^ This example motivates further optimization of BCP-templated materials through the control of chiral geometry, structural uniformity, orientation, and replication fidelity. The concept of templated chirality transfer could be further extended to selected portions of three-dimensional gyroid networks. Controlling nucleation and metal growth within these templates may provide a future route to finite chiral metallic motifs, whose replication fidelity and chiroptical response would need to be established.

### 6.2 Periodic Chiral Optical Networks

A conceptually distinct regime is reached in metallic single-gyroid metamaterials. Unlike finite Au nanohelices, the single gyroid is a continuous, periodically connected three-dimensional network. Wilts and co-workers fabricated Au and Ag single gyroid thin films as optical metamaterials through bottom-up replication of self-assembled triblock terpolymer templates (Figure 9a).^45^ The resulting metallic networks exhibit strong circular dichroism in the visible region, with the sign of the optical response determined by the chirality of the gyroid network. Under right- and left-circularly polarized illumination, the same silver gyroid film displays opposite contrast from neighboring microdomains with opposite chirality (Figures 9b and 9c), directly demonstrating the chirality-dependent interaction between circularly polarized light and the chiral gyroid lattice. This behavior is further confirmed by polarization-dependent transmittance spectra measured from an individual gyroid microdomain (Figure 9d), whereas CD spectra collected from multiple microdomains exhibit positive and negative mirror-image responses corresponding to gyroid networks with opposite chirality (Figure 9e). In such periodically connected structures, the chiroptical response reflects not only local plasmonic resonances but also collective electromagnetic coupling, Bloch-mode dispersion, and the symmetry and long-range periodicity of the gyroid lattice.^45,65–67^ Although these single-gyroid metamaterials exhibit strong chiroptical responses, the handedness of individual gyroid microdomains remains randomly distributed because self-assembly does not select a single macroscopic handedness. Instead, the net optical response depends on the population ratio of oppositely handed domains and may therefore vary among independently prepared samples. In contrast, homochiral evolution in chiral block copolymers can provide a preferred handedness in the polymer template. Combining such enantiomorphic gyroid templates with inorganic replication therefore provides a promising route toward enantiomerically defined three-dimensional plasmonic-photonic metamaterials. Studies of gyroid grain structure already offer useful approaches to template quality assessment. Gunkel and co-workers used confined crystallization to visualize large gyroid grains optically.^68^ Such characterization could potentially support grain-orientation assessment and quality control in chiral gyroid fabrication, alongside efforts to improve structural uniformity and metal replication fidelity.

**Figure 9:**
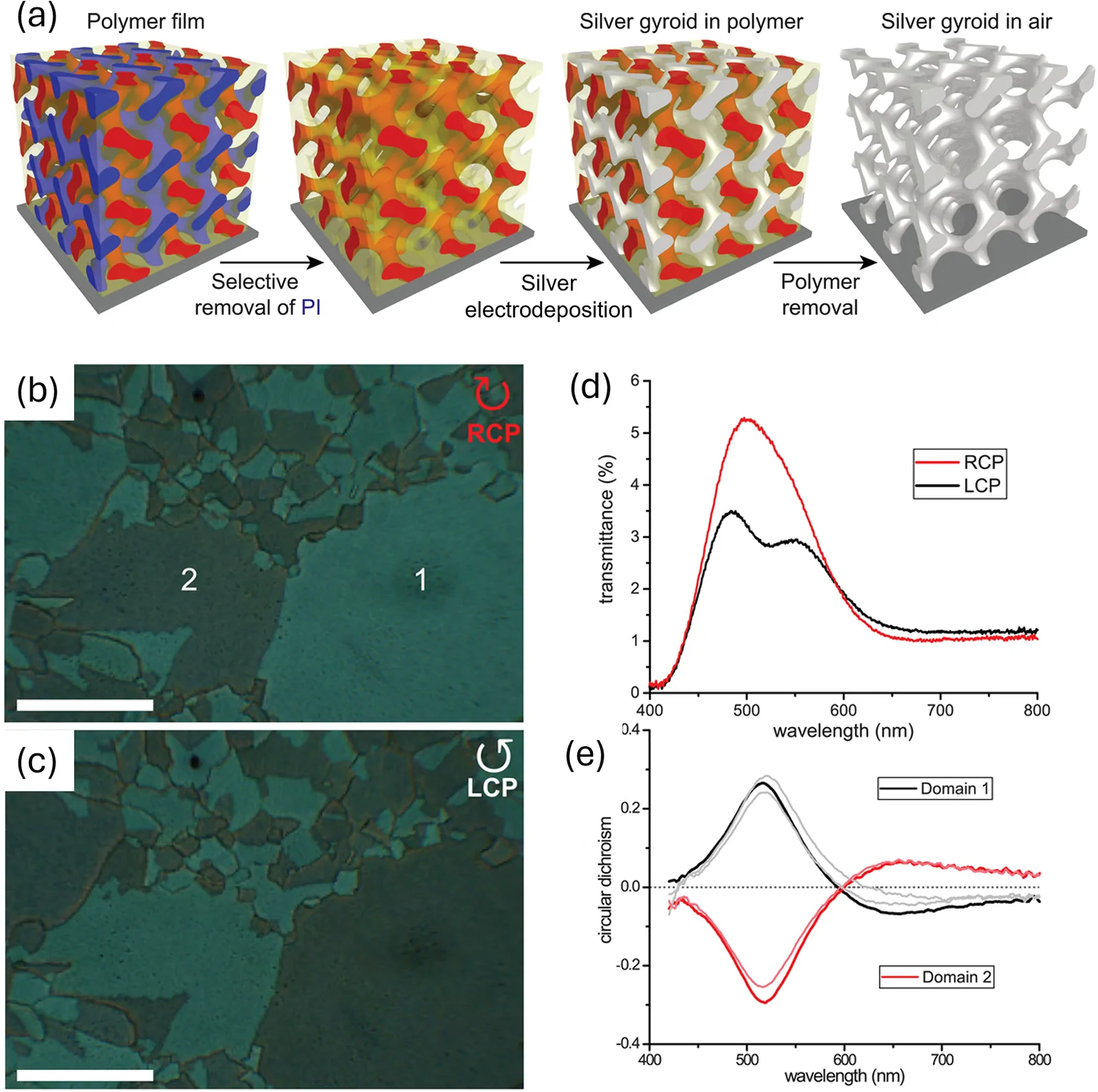
(a) Schematic illustration of the fabrication of a metallic single gyroid optical metamaterial through replication of a self-assembled triblock terpolymer template. (b,c) Optical microscopy images of a [110]-oriented silver single gyroid film under right- and left-circularly polarized illumination, respectively, revealing domains with opposite handedness through contrast inversion. (d) Polarization-dependent transmittance spectra recorded from an individual gyroid domain, showing distinct transmission for right- and left-circularly polarized light. (e) Circular dichroism spectra obtained from multiple domains, exhibiting positive and negative mirror-image responses associated with opposite-handed single gyroid networks. Scale bars in (b) and (c): 200 μm. Reproduced with permission from ref 45. Copyright 2020 Wiley-VCH GmbH.

## 7 Perspectives

Hierarchical chirality represents one of the most fascinating manifestations of molecular self-assembly, in which chirality evolves across multiple length scales from molecular building blocks to functional superstructures, giving rise to functional complexity in natural and synthetic materials. Among the self-assembled organizations, chiral block copolymers provide a unique model system for investigating this process because the corresponding molecular chirality, conformational chirality, supramolecular chirality, and phase chirality can all be systematically designed and characterized, establishing correlations within a single material platform; moreover, the development of novel chiral materials at the nanoscale is appealing and promising for creative applications. In this Viewpoint, we summarize recent advances in understanding homochiral evolution in block copolymer self-assembly and the resulting nanochiral phases, covering the fundamental mechanisms governing the emergence of phase chirality, experimental strategies for controlling chiral morphologies, methods for characterizing chirality at different length scales, and the translation of phase chirality into novel functional materials. These studies collectively demonstrate that block copolymer self-assembly is not only an excellent platform for exploring the fundamental principles of chirality transfer and amplification but also a versatile bottom-up strategy for constructing nanochiral phases with programmable architectures and optical functionalities for applications. Ultimately, understanding and controlling homochiral evolution in block copolymer self-assembly provide fundamental insights into the origin of hierarchical chirality and establish a general bottom-up paradigm for designing next-generation chiral functional materials.^5,13,15,43,45,53,62^

The characterization framework discussed in Section 3 provides the starting point for examining unresolved questions in homochiral evolution. While three-dimensional electron tomography directly determines the handedness of self-assembled phases with space-group symmetry arising from periodic ordering, it provides limited information regarding how molecular chirality is progressively transferred through polymer conformation and intermolecular organization. Building on the spectroscopic and structural correlations discussed in Section 4.3, a remaining challenge is to quantify how interchain chiral interactions connect single-chain conformation to phase handedness. Comparing isolated and interacting chain models, potentially using ab initio molecular dynamics, could help to identify and quantify how molecular packing contributes to VCD spectra; calculations on chiral molecular crystals provide a methodological precedent.^69^ Experimental comparisons with independently characterized conformation, crystallinity, and morphology would help to establish the packing-spectra relationship and connect these interactions to phase chirality.

The critical role of the microphase-separated interface with twisting and shifting or bending can be further supported by employing achiral chromophores as molecular probes. For instance, by incorporating perylene at the interface between achiral and chiral blocks, ICD of the perylene reveals that the chromophore adopts a one-handed helical arrangement directed by the chiral interface.^70,71^ More importantly, this interfacial chirality can be retained after selective removal of the chiral block, demonstrating a chiral memory effect and thus offering significant opportunities for chiroptic applications.^70^ The origin of this interfacial chirality has been further elucidated by systematically varying block sequence and interfacial interactions. Furthermore, introducing a chemically distinct midblock shows that the efficiency of cross-domain chirality transfer is governed primarily by the interaction between the chiral block and the intermediate microdomain, revealing that interfacial interactions may regulate the propagation of chirality across microphase-separated microdomains.^70–72^ Accordingly, the chemical junctions in block copolymers can be designed to tailor the effects of chirality on self-assembly. An unresolved question is how these interfacial interactions quantitatively influence phase handedness when composition and morphology are independently controlled.

Although molecular chirality can influence block copolymer self-assembly, the efficiency of chirality transfer appears to depend strongly on the physical characteristics of the constituent polymers. In particular, chain stiffness, commonly characterized by the persistence length, may play an important role in determining whether molecular chirality can be effectively amplified into higher-order structures. Semirigid polymers are expected to accommodate local chiral packing while remaining sufficiently deformable to undergo mesoscale structural rearrangements. In contrast, highly flexible polymers may not be able to sustain long-range orientational correlations, whereas excessively rigid chains may resist the collective bending or twisting required for the formation of nanochiral phases. Consequently, although chirality-induced self-assembly has been demonstrated in a wide range of polymer systems, successful homochiral evolution may occur only within an appropriate window of chain stiffness. Establishing quantitative relationships between persistence length and chirality transfer efficiency therefore represents an important direction for future theoretical and experimental investigations.

Building on the approaches discussed in Section 5, a key unresolved question is how induced conformational chirality can be translated into controlled phase handedness through interchain chiral interactions. Future comparisons can be carried out by examining the effects of dopant association and distribution while accounting for changes in segregation strength and effective block composition. Currently, the examination of the chirality effect on block copolymer self-assembly mainly focuses on the chiral block with main-chain chirality. It will be particularly interesting to study the effect of side-chain chirality on block copolymer self-assembly in terms of the formation of nanochiral phases.

With the formation of nanochiral phases such as the H* and G^A^, the self-assembly of chiral block copolymers creates opportunities beyond fundamental studies of symmetry breaking. Future progress will require a quantitative understanding of how chain stiffness, interfacial interactions, molecular architecture, and chiral interaction strength govern the efficiency of chirality transfer. Establishing these structure–property relationships will be essential for transforming homochiral evolution from an observed self-assembly phenomenon into a predictive design principle for next-generation chiral soft materials. For chiroptical applications of bulk and thin-film materials, these advances will need to be connected to reproducible optical performance, including transmission, dissymmetry, and optical loss under specified measurement conditions.

Block copolymer-templated synthesis can extend the applications of self-assembled nanochiral phases. Their well-defined handedness and nanoscale periodicity enable them to serve as templates for transferring chirality into inorganic and hybrid materials. Consequently, plasmonic nanohelices and periodic single-gyroid optical networks can be fabricated. Such hierarchical replication may provide scalable bottom-up routes toward circular-polarization-selective devices, chiral sensors, optical filters, and three-dimensional plasmonic-photonic metamaterials as well as chiral metasurfaces. Realizing these possibilities requires control of orientation, structural uniformity, and metal replication fidelity, together with demonstrations of consistent performance over representative areas and batches.
